# Human Bocavirus: Lessons Learned to Date

**DOI:** 10.3390/pathogens2010001

**Published:** 2013-01-11

**Authors:** Oliver Schildgen

**Affiliations:** Kliniken der Stadt Köln gGmbH, Krankenhaus Merheim, Klinikum der Privaten Universität Witten/Herdecke, Institut für Pathologie, Ostmerheimer Str. 200, D-51109 Köln (Cologne) Germany; E-Mail: schildgeno@kliniken-koeln.de; Tel.: +49-(0)221-8907-13467; Fax: +49-(0)221-8907-3542

**Keywords:** human bocavirus, HBoV, respiratory virus, gastrointestinal virus

## Abstract

Human bocavirus (HBoV) was identified as the second human parvovirus with pathogenic potential in 2005 in respiratory samples from children suffering from viral respiratory infections of unknown etiology. Since its first description, a large number of clinical studies have been performed that address the clinical significance of HBoV detection and the molecular biology of the virus. This review summarizes the most important steps taken in HBoV research to date and addresses open questions that need to be answered in the future to provide a better understanding of the role of a virus that is difficult to grow in cell culture and is suspected to be a pathogen, although it has not yet fulfilled Koch’s postulates.

## 1. Introduction

For a long time, the number of respiratory infections with clinical symptoms caused by respiratory viruses had no detectable causative agent. With the discovery of human metapneumovirus in 2001 by Bernadette van den Hoogen and her colleagues [1], virus discovery methods have become a focus of virologists, and there has been a marked increase in the number of newly detected viral pathogens. One of those pathogens is human bocavirus (HBoV), which was initially identified by Tobias Allander in 2005 [2] in 17 respiratory samples from children suffering from a respiratory tract disease of suspected viral origin.

Since the discovery of HBoV, numerous studies related to the epidemiology, pathogenesis, and replication of this newly detected virus have been performed, and they have provided novel insights into viral infection of the respiratory tract and gastrointestinal diseases.

## 2. Molecular Biology of HBoV

Based on sequence analyses of the 17 isolates identified by Allander and coworkers, HBoV was grouped into the family *Parvoviridae*, subfamily *Parvovirinae*, and genus *Bocavirus.* Currently, four subtypes have been identified, and of these, HBoV1 is most frequently detected in clinical samples of the respiratory tract. The remaining isolates are more frequently associated with gastrointestinal infections and symptoms [3,4,5,6].

The HBoV genome length ranges from 5,217 to 5,299 nucleotides [4] plus the terminal sequences, which are between 32 and 52 bases [7,8]. Based on phylogenetic analyses and electron microscopy analyses of the HBoV genomes from clinical specimens, the genome is believed to consist of a single-stranded DNA [9,10] of negative polarity. The negative polarity has been confirmed by real-time NASBA [11]. The terminal sequences, which are assumed to play a crucial role in the initiation of viral replication in the *Parvoviridae* family, have been identified as linker sequences in episomal genomes or replication intermediates in HBoV1 [7] and HBoV3 [8] and have been shown to have high homology to the terminal sequences of minute virus of canines (MVC) and bovine parvovirus [4], the latter providing a hint about the zoonotic origin of HBoV, either decades or centuries ago.

Recently, Zhao and colleagues showed that the genome of HBoV subtype 2 is circular [12]. This circular nature of the genome raises the question of why the hairpin structures are different between the HBoV subtypes, and it should be determined whether these linker structures are of cellular or other origin.

To date, the genome replication of HBoV has been assumed to occur in the same manner as the genome replication of animal parvovirus, that is, via a rolling hairpin mechanism, which would generate head-to-head or tail-to-tail intermediates, but the recently detected head-to-tail sequences do not fit with this replication model [7,8]. A novel cell culture system based on NuLi and CuFi cells, immortalized cell lines with the ability to differentiate in an air–liquid interface culture, could be appropriate models in which to study the questions regarding HBoV replication [13]. This recently published cell culture model is able to support HBoV replication and produce classical replication forms for parvoviruses; moreover, it was shown that the full-length HBoV-1 genome cloned into a plasmid vector was able to produce infectious particles in stable cells after transfection of the plasmid [13].

It appears likely that HBoV either persists or uses additional or alternative mechanisms of replication, which remains a matter of speculation.

An alternative replication model could be a helper virus-dependent initiation of rolling circle replication. In a recent case report (below), we identified herpesvirus 6 co-infection in an HBoV-infected patient. Surprisingly, during cidofovir therapy, HHV-6 viremia decreased, and HBoV was eliminated, even though the patient had an underlying immunodeficiency and could not produce neutralizing antibodies [14]. Considering that herpesviruses are able to initiate rolling circle replication [15] and replication that is characterized by head-to-tail sequences in *cis* and in *trans* [16], and given that another subfamily of parvoviruses, the dependoviruses, replicate exclusively in the presence of helper viruses, such as herpesviruses [17], this replication model is worth being discussed and investigated further.

The viral transcriptome has been analyzed in 2 studies, which revealed that RNAs for the proteins NS1, NP1, UP1/NP1, VP1/VP2, UP2/VP1/VP2, a putative ORFx protein, and an NS1-70 protein [18,19] are most likely transcribed. The genome organization is typical of a member of the *Bocavirus* genus. The viral genome contains at least three open reading frames (ORFs). Although it has not been experimentally verified for all encoded genes, it is widely assumed and likely that the proteins encoded by the HBoV genome are similar in function to the proteins of better characterized members of the *Bocavirus* genus. Thus, the first ORF at the 5' end likely encodes a nonstructural protein known as NS1, whereas the subsequent ORF likely encodes a second non-structural protein, NP1, that is believed to be unique to bocaviruses. The third ORF encodes the VP1 and VP2 proteins.

There is currently little information on the interactions of HBoV proteins with each other and with cellular proteins, primarily because secondary reagents, such as HBoV protein-specific antibodies and a versatile cell culture system, are not available. These materials are necessary for the investigation of such interactions. The novel reverse genetics system published by Huang and colleagues could be a useful tool for the analysis of the functions of HBoV proteins.

Overexpressed VP2 protein is able to form capsid-like structures that strongly resemble viral particles [20]. VP2 particles were used to analyze Th cell immunity by evaluating HBoV-specific T-cell proliferation in T-cells isolated from healthy adults [21]. In contrast to parvovirus B19, HBoV induces a less divergent Th response with respect to proliferation and interferon gamma, IL10 and IL13 production. The NP1 protein was shown to have immunomodulatory effects; using a cell culture approach with a nearly full-length clone of HBoV, Zhang and colleagues have shown that NP1 is able to indirectly block the IFN-β promoter and thus inhibit the production of interferon beta [22].

## 3. Epidemiology

HBoV is a major pathogen detected in respiratory and gastrointestinal infections. To date, four subtypes have been identified, and they are found worldwide, without any regional, geographic, or border restrictions. Following its initial discovery in Sweden, HBoV has been detected all over Europe [5,23,24,25,26,27,28,29,30,31,32], North [32,33,34] and South America [35,36,37,38,39], Africa [40,41], Asia [42,43,44,45], and Australia [46,47,48,49,50].

The four distinct subtypes have been named HBoV1–4. Subtype 1 is mainly associated with respiratory diseases, but can also be found in stool samples from patients suffering from diarrhea. The prevalence in symptomatic patients is approximately 1.5–16% for HBoV-1 [51,52] 21–26% for HBoV-2 [6], approximately 1% for HBoV-3 [53], and 0.6% for HBoV4 [54]. It appears that HBoVs have a high frequency of recombination among each other, as some subtypes are derived from recombinations of two others [6], and novel variants seem to occur more frequently than initially assumed [53].

The seroprevalence of HBoV is strongly dependent on the age of the investigated patient cohort and ranges from ~40% in children between 18 and 23 months of age up to virtually 100% in children older than two years, with an average of 76.6% in children and 96% in adults [55,56]. The seroprevalence is lowest for HBoV-4 (0.8–5%), followed by HBoV-3 (10–38.7%), HBoV-2 (34–49.3%), and HBoV-1 (66.9–96%) [55,57].

Recent clinical studies on respiratory infections that used novel multiplexing assays have shown that more severe infections (*i.e.*, infections that are clinically relevant, require hospitalization, and receive a proper laboratory diagnosis) frequently represent co-infections with up to six different pathogens in a single patient [51,52,58,59,60,61,62]. The range of co-infections or, more precisely, the rate of co-detection of pathogens that occur simultaneously with HBoV (or vice versa), ranges from 60 to 90%. This high rate can be explained by the fact that HBoV can be shed by asymptomatic patients and is able to persist [7,8,63], but it could also be the result of a better study design. During the last several years, it has become impossible to publish a study on respiratory viruses without screening for all viruses known at the time of the study. This requirement is one reason why the aforementioned studies used multiplexing technology and revealed marked frequencies of double or multiple infections (up to 44%) independent of the pathogens investigated [51,52,58,59,60,61,62]. Therefore, HBoV is not exclusively a bystander, but rather, the study cohorts investigated in the past (mainly hospitalized patients) suffered from multiple infections more frequently than previously assumed. 

## 4. Pathogenesis

In contrast to other viruses, from the first clinical studies until the present, HBoV has been co-detected with more additional pathogens than any other respiratory virus. This fact led to the hypothesis that HBoV may be a harmless bystander rather than a true pathogen, a hypothesis that was supported by the fact that it is impossible to fulfill Koch’s postulates for HBoV due to technical restrictions, i.e., currently, neither a versatile cell culture system nor an animal model has been established, nor have there been documented cases of the human-to-human transmission of HBoV.

There is currently limited information about the pathogenesis of HBoV. HBoV can be found in respiratory tract secretions [27] and stool [64,65,66]. HBoV also causes viremia (i.e., during active replication, the virus is detectable in the blood/serum of infected patients [55,67,68,69,70,71,72,73]). Furthermore, HBoV can be found in the duodenum [14], paranasal sinus mucosa [74], and intestinal biopsies [8]. Considering that in organ-specific air–liquid interface cell culture [18], HBoV particles were secreted both apically and distally, i.e., into the air phase and the liquid phase (which mimics the bloodstream), the most likely model for HBoV pathogenicity is a model analogous to that for minute virus of canines. This virus enters the host via the respiratory tract, reaches the bloodstream, and enters the gastrointestinal tract via the bloodstream or ingestion. Finally, viral shedding takes place via either coughing or defecation [75]. This model of pathogenesis is supported by the fact that the seroprevalence of HBoV is highest for the respiratory subtype HBoV1, whereas HBoV2–4 are less frequent and less seroprevalent [55].

Currently, it appears that all age groups can be affected by HBoV, although severe infections and infections requiring hospitalization occur primarily in patients with an underlying disease [76,77,78,79,80,81,82,83]. Severe clinical cases have been described in children [76,77], adults with cancer [76] and other risk groups [84]. 

## 5. Treatment

There is currently no specific approved treatment for HBoV infection [85]. Symptomatic treatment may be required in severe cases and is analogous to the treatment of other respiratory tract infections. To date, there has been only a single case report in which antiviral treatment was associated with the elimination of HBoV in a patient co-infected with HBoV and HHV-6 [14]. The boy suffered from an immunodeficiency and had lost the ability to mount an antibody-based immune response. During treatment with cidofovir, which is specific for herpesviruses, HHV-6 viremia decreased, and the HBoV infection was successfully eliminated. The outcome of this case is consistent with the outcome of another clinical case that we observed, in which HBoV viremia decreased during treatment, although this case had a fatal outcome for other reasons [86]. 

It must also be mentioned that, due to the lack of an animal model and a versatile cell culture system, the development of a vaccine is extremely difficult, and therefore, no results of such efforts have been published. The prevention methods for HBoV infection are analogous to those of other respiratory virus infections, and for laboratory research, this virus is “treated” in the same manner as other parvoviruses. The German National Committee for Biologic Safety (Zentralkommission für Biologische Sicherheit, ZKBS) has consequently classified this virus as a biosafety level 2 agent.

## 6. Diagnosis

Due to the lack of a cell culture system, the diagnosis of HBoV infection is exclusively based on molecular detection methods. Most laboratories currently use in-house PCR and real-time PCR assays targeting the NP-1, NS-1 or VP1/2 gene [3], but other nucleic acid-based detection methods for the diagnosis of HBoV have been described [11]. A number of commercially available approved multiplexing assays have been developed and brought to the market. Some of these assays also detect human bocavirus, including the Luminex RVP assay (Luminex, USA) and the RespiFinder assay (Pathofinder, the Netherlands). It appears likely that other assays to detect HBoV will be developed, although in many currently available assays, such as the FilmArray (Idaho Diagnostics, USA) and the RespID assay (Luminex, USA), this pathogen is neglected. Of note, for those laboratories that follow FDA rules, it is important to keep in mind that the Luminex RVP xTAG fast assay has received FDA clearance, whereas the other assays with FDA approval do not detect human bocavirus. 

To correctly diagnose an HBoV infection, it is necessary to screen clinical samples from the respiratory tract or stool samples (depending on whether the primary symptoms are respiratory or gastrointestinal) and a corresponding serum sample [31,67,68,69,70,72]. The latter is of great importance, as viremia is observed only during active infection [31,67,68,69,70,72,87,88], whereas HBoV can be shed by otherwise healthy patients, most likely due to persistent infection without viremia [87,88].

## 7. Conclusion

Although it was discovered several years ago, there are still more questions than answers related to human bocavirus. Despite an increasing and overwhelming amount of evidence that the virus is in fact a pathogen, Koch’s modified postulates have not yet been fulfilled for HBoV, primarily because a versatile cell culture system and an animal model are not available. Moreover, the virus is frequently detected as a co-pathogen, primarily because with the discovery of HBoV, a new era of clinical studies of respiratory infections has begun. In these studies, patients are screened for virtually all viruses known at the time the studies are performed. Currently, the primary challenge of the HBoV community is to develop a cell culture system or an animal model in which the virus can be propagated. The most recently published cell culture system using CuFi and NuLi cells could be such a model.

These tools are a prerequisite for the search for answers to open questions, including questions regarding effective antivirals and disinfectants and the molecular pathogenesis of HBoV. These tools will also help to determine whether HBoV is in fact a serious pathogen and not simply part of the harmless microbiome of its hosts.

Based on the currently available data, until HBoV can be proven to be a harmless bystander, I recommend that HBoV be treated as a pathogen, and thus, an inverse presumption of innocence should apply for HBoV.

If one tries to follow the history of virus discoveries during the last decade and has performed clinical studies in the field of respiratory infections, it is obvious that with the increasing number of identified pathogens, the range of diagnostic tools required in a clinical study has also increased. Thus, the more known viruses there are, the more diagnostic assays are demanded by investigators, clinicians, and peer reviewers of the manuscripts that describe those studies. It thus remains unknown whether HBoV is in fact a pathogen or whether the co-detection rate was high because clinical studies on HBoV infections were among the first generation of studies in which the presence of virtually all respiratory pathogens was required to be assessed.
